# Correlation of Ocular Surface Disease and Quality of Life in Indian Glaucoma Patients: BAC-preserved versus BAC-free Travoprost

**DOI:** 10.4274/tjo.galenos.2019.29000

**Published:** 2020-04-29

**Authors:** Suresh Kumar, Tanu Singh, Parul Ichhpujani, Sanchi Vohra, Sahil Thakur

**Affiliations:** 1 Government Medical College and Hospital, Department of Ophthalmology, Sector 32B, Chandigarh, India

**Keywords:** Primary open-angle glaucoma, OSDI, BAC, travoprost, GQL-15

## Abstract

**Objectives:**

The use of benzalkonium chloride (BAC)-preserved medications is associated with ocular surface disease (OSD) that can negatively affect quality of life (QoL) in glaucoma patients. This study aimed to compare QoL and correlate it with OSD in glaucoma patients receiving BAC-preserved and BAC-free travoprost.

**Materials and Methods:**

A total of 110 subjects were divided into 3 groups: 40 primary open-angle glaucoma (POAG) patients using BAC-preserved travoprost, 40 POAG patients using BAC-free travoprost, and 30 age-matched controls. All patients were assessed using a single interviewer-administered format of the Ocular Surface Disease index (OSDI) and Glaucoma Quality of Life-15 (GQL-15) questionnaires.

**Results:**

Mean GQL-15 score in the BAC group was significantly higher than in the BAC-free group (24.71±7.42 vs. 17.58±3.06; p<0.05). The mean difference in GQL-15 scores between controls and the BAC-free group (1.24) was insignificant (p>0.05). There was a strong positive correlation between OSDI scores and GQL-15 scores in all the groups (r values: BAC: 0.63, BAC-free: 0.23, controls: 0.29), with higher OSDI scores (severe OSD) associated with higher GQL-15 scores (worse QoL). Cronbach’s alpha was 0.84 for GQL-15 and 0.75 for OSDI.

**Conclusion:**

BAC-preserved travoprost leads to higher OSDI scores, which correlate strongly with poor QoL scores as compared to BAC-free travoprost. The use of BAC-free formulations should be encouraged to reduce the onset or worsening of OSD and impaired QoL in glaucoma patients.

## Introduction

Quality of life (QoL) refers to the perceived quality of an individual’s daily life, that is, an assessment of their well-being.^1^ It is an assessment of how the individual’s well-being may be affected over time by a disease. Assessment of QoL is important in the management of glaucoma patients, as it reflects the patient’s own perception regarding the burden of a chronic disease. Therefore, in recent times, apart from traditional measures such as visual acuity, intraocular pressure (IOP) and perimetry, assessment of QoL is being increasingly recognized as a critical measure for evaluating the effectiveness of treatment of glaucoma.

Ocular surface disease (OSD) is a common co-morbidity in glaucoma, affecting around 59% of glaucoma patients.^2^ In addition, approximately 36% of these glaucoma patients have significant OSD requiring some form of treatment.^2^ Studies have shown that all classes of topical IOP-lowering medications can cause ocular surface discomfort and significant ocular surface changes with long-term use.^2,3,4^ The higher incidence of OSD in glaucoma patients is largely attributed to the use of benzalkonium chloride (BAC)-preserved topical antiglaucoma medication.^4,5,6^ OSD often negatively affects the patient’s ability to work and function, and therefore contributes to worsening of QoL in glaucoma patients. Since OSD can dramatically impact patients’ QoL, it influences therapeutic compliance and adherence to treatment.

The Ocular Surface Disease index (OSDI), developed by the Outcome Research Group (Irvine, CA),^7^ is a 12-item questionnaire providing a rapid assessment of the symptoms of ocular irritation related to OSD and their impact on vision-related functioning. It has been found to be a reproducible, reliable and valid tool for the assessment of OSD in glaucoma patients.^8,9,10^

Several disease-specific instruments have been used to assess QoL in glaucoma patients.^11,12,13^ The Glaucoma Quality of Life-15 (GQL-15) questionnaire is a disease-specific, 15-item questionnaire which has been shown to be reliable and have good internal consistency.^11^ Four important domains tested in this instrument are central/near vision, peripheral vision, night vision and outdoor mobility.

Glaucoma patients treated with BAC-preserved medications often have concomitant OSD leading to worsening of their QoL. Exposure to high daily dose of BAC-containing formulations was associated with even poorer quality of life.^14^ After a thorough literature search, we found a paucity of data regarding OSD and its impact on the QoL of glaucoma patients. In recent years, BAC-free antiglaucoma formulations have become commercially available.^15,16^ Some studies have reported that switching from BAC-containing to BAC-free antiglaucoma medication resulted in improvement in QoL scores using the Glaucoma Symptom scale (GSS).^17,18^ The use of BAC-free formulations reduce the onset or worsening of OSD and QoL in glaucoma patients.However, no study has compared and correlated QoL scores with OSD in BAC-preserved and BAC-free medications. The present study was designed to assess QoL using the GQL-15 questionnaire and determine its correlation with OSD according to the OSDI in glaucoma patients receiving BAC-preserved and BAC-free travoprost.

## Materials and Methods

The present research study was registered with the clinical trial registry of India, CTRI (CTRI/2017/12/011044CTRI) and was approved by the institutional ethics committee. It conformed to the tenets of the Declaration of Helsinki and followed HIPPA (Health Insurance Portability and Accountability Act). It was a hospital-based prospective observational study carried out in 110 subjects who visited the Glaucoma Services. Study subjects were divided into 3 groups: the first group comprised 40 primary open-angle glaucoma (POAG) patients using BAC-preserved travoprost and the second group included 40 POAG patients receiving BAC-free travoprost. A third group of 30 age-matched subjects not receiving any topical medical treatment were recruited as controls. To ensure uniformity, we considered only a single prostaglandin analogue and included BAC-free drugs with only polyquad (polyquaternium-1) as a preservative. The following inclusion and exclusion criteria were considered for subject selection.

**Inclusion Criteria:** The study included POAG patients over 40 years of age of either gender, with mild to moderate glaucoma (according to Hodapp Parrish Anderson classification), who had well-controlled IOP on prostaglandin monotherapy (BAC-preserved or BAC-free) for a minimum period of 3 months. The duration of therapy was set as a maximum period of 6 months. Appropriate history was taken to rule out use of any other topical hypotensive agent prior to the institution of prostaglandin therapy.

**Exclusion Criteria:** Patients with pre-existing OSD (anterior or posterior blepharitis, keratitis, ocular dryness, follicular or papillary conjunctivitis) prior to initiation of antiglaucoma medical therapy, corneal abnormalities which might preclude the calculation of IOP by applanation tonometry, prior refractive eye surgery, prior filtration surgery, allergic reaction to prostaglandin analogues, pregnant or lactating females, patients using contact lenses within 3 weeks of enrollment, and patients with other visually significant diseases like cataract, diabetic retinopathy, hypertensive retinopathy, age-related macular degeneration, which might act as confounding factors causing lower QoL were excluded from the study. Patients with cognitive, hearing, or mobility impairment which precluded appropriate response to the questionnaires were also excluded from the study. Patients with glaucoma grade beyond moderate glaucoma were excluded from the study to ensure that the severity of glaucoma did not act as a confounding factor in the evaluation of the subjects’ QoL.

**Baseline Examination:** All patients enrolled in the study underwent extensive ophthalmologic examination which included measurement of visual acuity by Snellen chart both with and without correction at a distance of 6 meters and detailed slit-lamp examination. Ocular examination was done to assess the lids (especially margins), palpebral and bulbar conjunctiva, cornea, pupillary reactions, and anterior segment, as well as posterior segment examination with +90D lens after pharmacological dilation. The IOP was measured at the time of enrollment with the help of a calibrated Goldmann applanation tonometer (GAT). Gonioscopy was performed on each patient with the help of a goniolens (Goldmann one-mirror lens as well as Zeiss four-mirror lens). Pre-enrollment assessments specific for ruling out OSD such as Schirmer’s test, tear film break-up time (TBUT), and fluorescein staining were done. Corneal fluorescein staining was graded as mild (less than 10% coverage of corneal surface), moderate (10-50% of corneal surface), and severe (more than 50% of corneal surface) and subjects with moderate or severe staining were excluded from the study. Central corneal thickness was measured using optical coherence biometer (Haag Streit, Lenstar). Visual field analysis was done with Humphrey visual field analyzer using HFA-24-2 Swedish Interactive Thresholding Algorithm (SITA) Fast 24-2 testing algorithm.

Primary open-angle glaucoma was defined as the presence of glaucomatous optic nerve head changes, open anterior chamber angles on gonioscopy, reproducible and reliable visual field on Humphrey Field Analyzer, with or without elevated IOP, in one or both eyes.

Detailed history of presence or absence of BAC, the duration since initiation of therapy, and treatment compliance was taken. The subjects found to fulfill all the inclusion criteria were asked to provide written informed consent. Subjective tolerance of the drug and QoL assessment was done using the interviewer-administered format of the OSDI and GQL-15 questionnaires, respectively. A single investigator administered both questionnaires to all the subjects at 2 different follow up visits (first after completion of 3 months of topical therapy and second at completion of 6 months). For respondents who were not literate in English, the OSDI and GQL-15 questionnaires were translated into Hindi and Punjabi, which are the most important vernacular languages in northern India.

### Ocular Surface Disease Index (OSDI) Questionnaire

The OSDI questionnaire was used to evaluate subjective tolerability of the drug affecting the ocular surface in all subjects. Each of the 12 items of OSDI questionnaire was graded on a scale of 0 to 4: 0=none of the time; 1=some of the time; 2=half the time; 3=most of the time; and 4=all the time. The total OSDI score is calculated using the following formula: OSDI = (Sum of OSDI item scores x 100)/(Total number of questions answered x 4).

OSDI score ranged from 0 to 100. The patients were categorized based on the scores as follows: normal ocular surface (0-12 points), mild OSD (13-22), moderate OSD (23-32), and severe OSD (33-100).

### Glaucoma-Associated Quality of Life (GQL-15) Questionnaire

The GQL-15 questionnaire was utilized to assess QoL in all patients. GQL-15 questionnaire is composed of 15 items which address 4 factors of visual disability: central and near vision (2 questions), peripheral vision (6 questions), dark adaptation and glare (6 questions), and outdoor mobility (1 question). A scale of 0 to 5 is used to code the item-level responses for each factor, where 5 represents severe difficulty due to visual reasons, 1 indicates no difficulty with performing the activity, and 0 indicates abstinence from activity due to non-visual reasons. Item scores were added to obtain a total score. Higher GQL-15 scores indicate poorer QoL. Subscale scoring for the abovementioned 4 domains of the GQL-15 was not done in our study.

### Statistical Analysis

Descriptive analysis was carried out by mean and standard deviation for quantitative variables and with frequency and proportion for categorical variables. The association between categorical explanatory variables and quantitative outcome was assessed by comparing the mean values. The mean differences along with their 95% confidence interval (CI) were presented. Independent-samples t-test/ANOVA/paired-samples t-test was used to assess statistical significance. The association between explanatory variables and categorical outcomes was assessed by cross-tabulation and comparison of percentages. Odds ratio along with 95% CI is presented. Chi-square test was used to test statistical significance. P value <0.05 was considered statistically significant. Pearson’s correlation was used to measure the linear correlation between OSDI and GQL-15 scores. Pearson’s correlation coefficient was mentioned as R-values; which ranged from +1 to -1, where +1 is total positive linear correlation, 0 is no correlation and -1 is total negative correlation. Cronbach’s alpha was used to assess the internal consistency of the two questionnaires. IBM SPSS version 22 was used for statistical analysis.

## Results

Mean age was 60.88±8.48 years in the BAC group, 61.25±13.32 years in the BAC-free group, and 60.42±7.16 years in the control group. The difference in mean age between the groups was statistically insignificant (p=0.45). The proportion of males was 60%, 62.5%, and 60% respectively in the BAC, BAC-free, and control groups. The differences in the gender composition of participants among the groups was statistically not significant (p=0.51). The time taken for administration of the OSDI questionnaire was about 5-6 minutes, while the GQL-15 questionnaire required 7-8 minutes. The mean IOP (in mmHg) at the time of enrollment was 12.3±3.8 in the BAC group, 11.9±2.9 in the BAC-free group, and 10.9±3.1 in the control group and the difference between the three groups was statistically insignificant (p=0.50). The mean central corneal thickness (in microns) in the three groups was 545±30, 541±25, and 550±12, respectively. The difference amongst the three groups was statistically insignificant (p=0.15). Compliance to treatment was determined based on history and was found appropriate as per the discretion of the examiner. While assessing the correlation of GQL-15 scores with visual function, GQL-15 scores correlated best with mean deviation in both eyes (MD OU) (r=0.61, p=0.001) and mean logMAR visual acuity in the worse eye (r=-0.41, p=0.001). Only a modest or weak correlation existed between pattern standard deviation and GQL-15 (r=0.11, p=0.14). Cronbach’s alpha was 0.84 for GQL-15 and 0.75 for OSDI.

Comparison of mean GQL-15 scores across the study groups: Mean GQL-15 score was 24.25±7.42 in the BAC group, 17.58±3.06 in the BAC-free group, and 16.33±1.92 in the control group. The mean difference between the BAC and BAC-free groups (6.68) was statistically significant (p=0.047). The mean difference between the BAC group and controls (7.92) was also statistically significant (p=0.042). The mean difference between the BAC-free group and controls (1.24) was not statistically significant (p=0.057) (Table 1).

Comparison of mean OSDI scores across the study groups: Mean OSDI scores in the BAC, BAC-free, and control groups were 29.09, 12.45, and 10.93, respectively. The mean difference between the BAC-free and BAC groups (16.63) was statistically significant (p<0.01). The mean difference between the BAC-free group and controls (1.53) was statistically insignificant (p=1.0). The mean difference between the BAC group and controls (18.96) was statistically significant (p<0.01) (Table 2).

There were only 23.33% subjects among controls and 32.5% in the BAC-free group with OSD, whereas the proportion of patients in the BAC group with OSD was 82.5%. The association between the groups and OSD was statistically significant (p<0.01).

**Figure 1:**
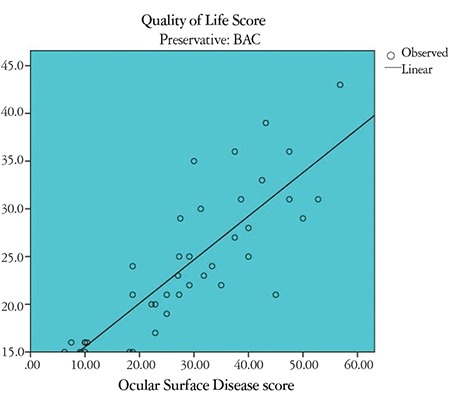
Scatter diagram showing correlation between Ocular Surface Disease index (OSDI) score and Glaucoma Quality of Life-15 (GQL-15) score in the BAC group

**Figure 2:**
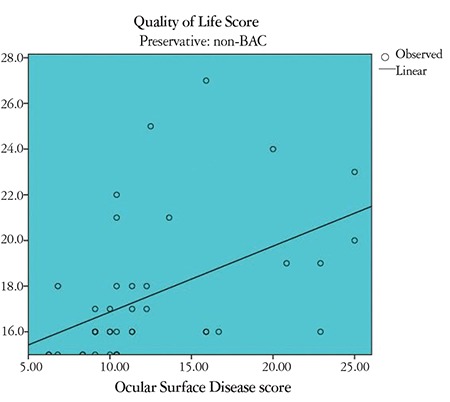
Scatter diagram showing correlation between Ocular Surface Disease index (OSDI) score and Glaucoma Quality of Life-15 (GQL-15) score in the BACfree group

Correlation between OSDI and GQL-15 scores in the study groups: There was a strong positive correlation between OSDI and GQL-15 score in the BAC group (r=0.78, p<0.01) (Figure 1). There was also a strong positive correlation between OSDI and GQL-15 score in the BAC-free group (r=0.64, p<0.01) (Figure 2). There was a highly significant, strong positive correlation between OSDI score and GQL-15 score among controls (r=0.58, p<0.001). Table 3 shows the correlation between OSDI and GQL-15 scores in the 3 groups.

## Discussion

The purpose of glaucoma treatment is to maintain the patient’s visual function and its related QoL. Glaucoma patients’ QoL can be considerably affected by the side effects of long-term use of antiglaucoma drugs.^14,16^ The present study confirmed that more severe OSD (higher OSDI scores) had a strong correlation with worse QoL (higher GQL-15 scores) in patients on BAC-preserved prostaglandins and less severe OSD (lower OSDI scores) had strong correlation with better QoL (lower GQL-15 scores) in patients on BAC-free prostaglandins.

As a preservative, BAC has detergent-like activity which has a propensity to compromise the tear film and induce or worsen pre-existing OSD.^19^ The prevalence of ocular symptoms such as discomfort upon instillation, burning/stinging, foreign body sensation, dry eye sensation, and tearing have been found to be more prevalent with BAC-preserved than with unpreserved antiglaucoma drugs (p<0.001).^20^ Switching from BAC-preserved to BAC-free medication led to a decrease in OSD in previous studies.^21,22^ In our study, the incidence of OSD (OSDI scores more than 12) was significantly higher in the BAC group than in the BAC-free group (82.5% versus 32.5%), similar to other studies. BAC is known to cause corneal toxicity and has a deleterious effect on TBUT.

In our study, there was a statistically strong correlation between OSDI scores and QoL scores among all the study groups. Mean QoL scores (16.1±2.3) on the GQL-15 questionnaire were found to be lower in patients having no OSD (OSDI score <12), whereas mean QoL scores (21.2±10.4) were found to be higher in patients having OSD (OSDI scores more than 12). The increase in mean QoL scores in patients with higher OSDI scores signifies lower QoL in these patients.

In our study, BAC-preserved antiglaucoma medication was associated with higher OSDI scores (18.72±7.04), which strongly correlated with poorer QoL (24.25±7.42) in these patients. In contrast, the use of BAC-free antiglaucoma medication was associated with lower OSDI scores (11.2±5.15), which correlated strongly with better QoL (17.58±3.06) in these patients. The study by Skalicky et al.^14^ showed a positive correlation between OSDI scores and QoL in glaucoma patients, similar to our study.Similarly, Rossi et al.^18^ concluded in their study that the presence of dry eye disease as assessed by the OSDI negatively influences the patient’s QoL.Glaucoma is known to adversely affect QoL and the presence of concomitant OSD further worsens QoL in these patients.

The mean QoL score in the BAC group (24.25±7.42) was significantly higher than in the BAC-free group (17.58±3.06, p<0.05), indicating poorer QoL in these patients. The worsened QoL can be attributed to the presence of more severe OSD in the BAC-preserved (18.72±7.04) versus the BAC-free group (11.2±5.15; p<0.001). Previous studies have documented higher incidence of OSD with use of BAC-containing antiglaucoma medications,^20,21^ while BAC-free medications which incorporate safer preservatives such as polyquaternium-1 (Polyquad^®^) were shown to reduce the incidence and severity of OSD.^15,16^ In a prospective study, Lester et al.^23^ used the GSS and found that changing BAC-preserved antiglaucoma drugs with BAC-free treatment improved QoL in glaucoma patients. In another prospective study by Abegao et al.^17^ using the same questionnaire (GSS), it was found that switching to BAC-free antiglaucoma drugs significantly improved self-reported QoL in glaucoma patients (p<0.001). Both of these studies used the GSS, which does not take into account the symptoms related to glare and peripheral vision.^24^ Therefore, it may underestimate difficulty experienced by patients in their day-to-day activities, especially driving at night. Our study found the BAC-free formulation to be superior to the BAC-preserved formulation in improving OSD-related QoL in glaucoma patients. We found that domains involving night vision on GQL-15 strongly correlated with QoL in these patients. GQL-15 has been shown to strongly correlate with both visual disability and psychophysical measures of visual functions. This is the first study to use the GQL-15 questionnaire for comparing QoL in BAC-preserved and BAC-free antiglaucoma medication.

In our study, the BAC-preserved group showed significantly higher QoL scores (p<0.05) than controls, indicating worse QoL in these patients. Similarly, Skalicky et al.^14^ in their study found that increasing glaucoma severity and higher exposure to BAC was associated with poorer QoL compared to controls (p<0.001). Another study using the Dry Eye Questionnaire 5 (DEQ5) and the Impact of Dry Eye on Everyday Life (IDEEL) questionnaire concluded that a greater number of glaucoma medications was associated with increased frequency of severe dry eye symptoms and decreased emotional QoL.^25^ In our study, we found that use of BAC-preserved antiglaucoma medication increased OSDI scores and led to lower QoL in glaucoma patients. However, the patients in our study were on monotherapy as opposed to multiple drugs in the previous study. These symptoms can have a substantial impact on a patient’s QoL. Our study proves that the use of BAC-preserved antiglaucoma drugs is associated with more severe OSD, which leads to worsened QoL in these patients.

There is paucity of literature which compares QoL in patients receiving BAC-free antiglaucoma drugs with controls. Rolle et al.^26^ recently reported that patients using unpreserved tafluprost and unpreserved timolol had significantly worse OSDI and QoL scores than controls (p=0.000), thereby implying the possible role of the active ingredient in reducing QoL in these patients. However, in our study QoL was found to be statistically similar in the BAC-free and control groups. Therefore, from our study it is evident that QoL was not significantly affected by BAC-free topical medications. OSD-related poor QoL in glaucoma patients can be largely attributed to BAC, and the use of BAC-free prostaglandins has the potential to improve QoL in these patients.

### Study Limitations

One limitation in the design of the present study was that it was a non-randomized study, which may have resulted in selection bias. We included patients exclusively with mild to moderate glaucoma and hence comparison of QoL could not be made between different grades of glaucoma severity. Our data relies on patient-reported surveys which can be influenced by recall bias. The strong points of our study are that we used valid, reliable, and disease-specific instruments like GQL-15 and OSDI questionnaires for all patients and the questionnaires were administered by a single interviewer, thus eliminating personal bias. Our study had a good sample size that included an adequate number of patients in all the study groups.

## Conclusion

There is a strong correlation between OSD and QoL in medically treated glaucoma patients. The increasing severity of OSD correlated with worsening of QoL. BAC-preserved prostaglandins were associated with significantly higher OSD and worse QoL in glaucoma patients compared to BAC-free travoprost. Patients using BAC-free travoprost had statistically similar OSD and glaucoma-related QoL to controls. Therefore, shifting to BAC-free antiglaucoma medication is recommended for improving OSD-related QoL in these patients.

## Figures and Tables

**Table 1:**
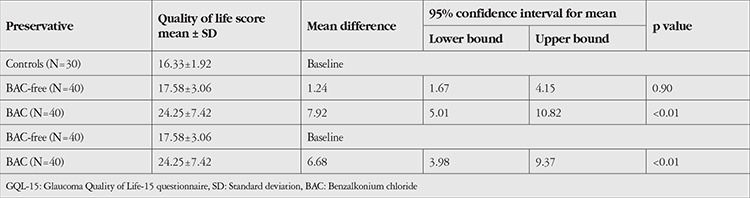
Comparison of mean GQL-15 scores

**Table 2:**
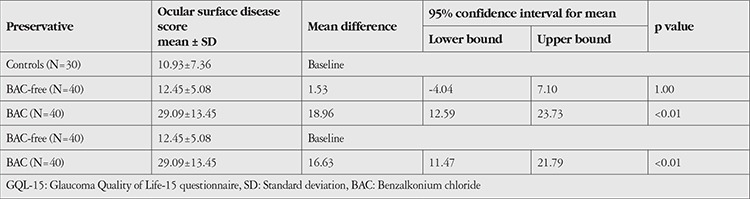
Comparison of mean OSDI scores

**Table 3:**
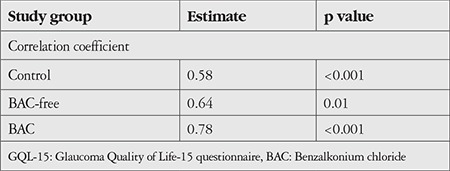
Correlation between OSDI and GQL-15 scores in the study groups
